# A framework for outcome-level evaluation of in-service training of health care workers

**DOI:** 10.1186/1478-4491-11-50

**Published:** 2013-10-01

**Authors:** Gabrielle O’Malley, Thomas Perdue, Frances Petracca

**Affiliations:** 1 International Training and Education Center for Health, Department of Global Health, University of Washington, 901 Boren Avenue, Suite 1100, Seattle, WA 98104, USA

**Keywords:** Health worker training, Training evaluation framework and tools, Training outcomes

## Abstract

**Background:**

In-service training is a key strategic approach to addressing the severe shortage of health care workers in many countries. However, there is a lack of evidence linking these health care worker trainings to improved health outcomes. In response, the United States President’s Emergency Plan for AIDS Relief’s Human Resources for Health Technical Working Group initiated a project to develop an outcome-focused training evaluation framework. This paper presents the methods and results of that project.

**Methods:**

A general inductive methodology was used for the conceptualization and development of the framework. Fifteen key informant interviews were conducted to explore contextual factors, perceived needs, barriers and facilitators affecting the evaluation of training outcomes. In addition, a thematic analysis of 70 published articles reporting health care worker training outcomes identified key themes and categories. These were integrated, synthesized and compared to several existing training evaluation models. This formed an overall typology which was used to draft a new framework. Finally, the framework was refined and validated through an iterative process of feedback, pilot testing and revision.

**Results:**

The inductive process resulted in identification of themes and categories, as well as relationships among several levels and types of outcomes. The resulting framework includes nine distinct types of outcomes that can be evaluated, which are organized within three nested levels: individual, organizational and health system/population. The outcome types are: (1) individual knowledge, attitudes and skills; (2) individual performance; (3) individual patient health; (4) organizational systems; (5) organizational performance; (6) organizational-level patient health; (7) health systems; (8) population-level performance; and (9) population-level health. The framework also addresses contextual factors which may influence the outcomes of training, as well as the ability of evaluators to determine training outcomes. In addition, a group of user-friendly resources, the Training Evaluation Framework and Tools (TEFT) were created to help evaluators and stakeholders understand and apply the framework.

**Conclusions:**

Feedback from pilot users suggests that using the framework and accompanying tools may support outcome evaluation planning. Further assessment will assist in strengthening guidelines and tools for operationalization.

## Background

There is widespread recognition that the lack of an adequately trained health care workforce is a major barrier to scaling up and sustaining health-related services in resource-limited settings worldwide [1]. In-service training for health care workers has proliferated as a key strategic approach to this challenge, particularly in response to the HIV/AIDS epidemic. The United States President’s Emergency Plan for AIDS Relief (PEPFAR) alone supported close to four million training and re-training encounters between 2003 and 2008 [2]. Between 2002 and mid 2012, programs supported by the Global Fund to Fight AIDS, Tuberculosis and Malaria provided 14 million person-episodes of training [3].

These numbers reflect the widely accepted understanding that well-trained, well-prepared health care workers enable stronger health systems and better patient health. Despite commitment to these goals, however, many of the largest international programs supporting in-service training do not consistently require or provide evidence linking specific training efforts to their desired outcomes. Rather, programs generally report what are commonly referred to as training “outputs,” such as the number of people trained, the professional category of person trained and the training topic [4-6]. These output indicators enable funders, governments and training program staff to aggregate implementation data across a variety of topic areas and types of training encounters (for example, workshops, lectures, distance education and long-term mentoring). However, output indicators do not help evaluate how well the training encounters are improving provider practice or patient health outcomes. Recently, there has been a renewal of concerns raised about the lack of evidence linking the resources invested in health care worker in-service trainings to improved health outcomes [7-10].

However, this call for more frequent and rigorous evaluation linking clinical and public health training to provider performance and patient outcomes is not new. Nor are the challenges to implementing such evaluations. A 2001 review of 599 articles from three leading health education journals revealed that trainer performance (teaching skill) and trainee satisfaction were the most commonly identified types of training evaluations. However, neither measure necessarily reflects improvements in patient care [11]. In the same year, a broader review of health professional behavior change interventions published between 1966 and 1998 found incomplete but valuable insights into the likely effectiveness of different training interventions [12]. The reviewers identified the difficulty of disentangling which components of multifaceted interventions were likely to be effective and complementary under different settings. Methodological issues have also been reported as especially challenging for identifying results arising from training evaluations. These include the distal nature of outcomes and impacts from the training (training as a necessary but insufficient condition) [7], the number of confounders [7,13], the lack of easily generalizable findings due to the singular nature of different learning and practice environments [1,14,15] and the lack of funding dedicated to evaluation [13,14].

In spite of these challenges, it remains important to evaluate the effectiveness of training. Such evaluation ensures that increasingly limited financial resources, and the hours that health care workers devote to attending in-service training, are money and time well spent.

Multiple frameworks have been developed to guide managers, evaluators and policy makers as they think about how to evaluate the complex and highly variable phenomenon commonly referred to as “training.” The most frequently referenced training evaluation framework is the Kirkpatrick Model, which was designed primarily for use in business and industry and has been in broad use for over half a century [16,17]. The model identifies four levels at which trainings can be evaluated: Reaction, Learning, Behavior and Results. It has been critiqued, refined and adapted for various purposes, including evaluations of military training [18], leadership training [19] and workplace violence prevention training [20]. One integrated model for employee training combines a Kirkpatrick-based evaluation of training outcomes with a novel approach for understanding how and why those outcomes occur [21]. Each framework offers valuable insights to support evaluation planning.

Recognizing that it is important to demonstrate the outcomes of substantial investments in health worker training, but that existing evaluation models may not provide theoretical and practical resources that can be readily applied to HIV and AIDS training programs, the PEPFAR Human Resources for Health Technical Working Group initiated a project to develop an outcome-focused training evaluation framework. The purpose of the framework is to provide practical guidance for health training programs in diverse international settings as they develop their approaches to evaluation. This paper presents the methods and results of that project.

## Methods

The framework was conceptualized and developed in three steps: 1) Data collection; 2) Data analysis and initial framework development; and 3) Refinement and validation of the framework through an iterative process of feedback and revision.

All of the methods used in the steps align with an overarching inductive approach [22]. The inductive approach seeks to identify themes and categories in qualitative data, in order to develop a “model or theory about the underlying structure of experiences or processes that are evident in the text data” [22]. The approach was well-suited to the work of translating diverse qualitative information on in-service training outcome evaluation into a structured, responsive and meaningful framework.

### Data collection

Data were collected through two main activities. Key informant interviews were used to explore the broad context in which evaluation of training outcomes takes place; the perceived value of evaluation; and needs and barriers. In addition, a thematic analysis of published articles reporting health care worker training outcomes was conducted.

#### Key informant interviews

Between June 2011 and December 2011, key informant interviews were conducted with health care worker training program managers and staff members, PEPFAR-funded program directors and technical advisors, PEPFAR Human Resources for Health Technical Working Group members, administrators from the Office of the U.S. Global AIDS Coordinator, and other key stakeholders. Convenience sampling was initially used to identify potential respondents working in capacity building for global health programs. Subsequent snowball sampling resulted in a total of 15 key informants who had direct programmatic, management or technical support experience with health programs engaged in training and/or training evaluation. Face-to-face interviews were conducted by three experienced interviewers, using a semi-structured approach with an open-ended interview guide. The guide included the following topics:

•Perceptions of the current state of training evaluations

•The evolving need for in-service training evaluation

•Need for technical assistance to programs around in-service training evaluation

•Best approaches to obtaining outcome evaluation data

•Barriers and facilitators to obtaining outcome-level data

•Extent to which health outcomes can be attributed to training interventions

•Practical uses for outcome evaluation findings

•Existing resources for supporting in-service training outcome evaluation

Deviations from specific topics on the interview guide and tangential conversations on related topics allowed key informants the flexibility to prioritize the issues and themes they deemed important based on their personal and professional perspectives. The interviews lasted between 40 minutes and 2 hours, and were digitally recorded. Additionally, interviewers took written notes during the interviews to identify and expand on important points.

#### Thematic analysis of published articles

A thematic analysis of published articles reporting health care worker training outcomes provided information on the range of training evaluations in the peer reviewed literature, specifically the type of training outcomes the authors chose to evaluate, and the methodological approaches they used.

This process followed an inductive approach to qualitative analysis of text data [23]. This methodology differs from a standard literature review in that its primary purpose is not to exhaustively examine all relevant articles on a particular topic, but rather to identify a range of themes and categories from the data, and to develop a model of the relationships among them.

Similar to the approach described by Wolfswinkel [24], a focused inquiry initially guided the search for articles. This was followed by a refining of the sample as concurrent reading, analysis and additional searching were conducted. To identify the data set of articles for thematic analysis, the team searched multiple databases for articles on health training and evaluation (PubMed, MANTIS, CINAHL, Scopus) for the period 1990 to 2012 using the key search terms “training,” “in service,” “health systems” and “skills,” combined with the terms “evaluation,” “impact,” “assessment,” “improvement,” “strengthening,” “outcomes,” “health outcomes” and “health worker.” Three reviewers collected and read the articles that were initially retrieved. Additional articles of potential interest that had not appeared in the database search but were identified in the reference sections of these papers were also retrieved and reviewed. Articles were included if they reported study findings related to training interventions for professional health care workers or informal health care workers, such as traditional birth attendants and family caregivers. Training interventions included both in-person and distance modalities, and ranged from brief (for example, one hour) to extended (for example, one year) training encounters. Review articles were excluded, as were articles reporting methods but not results, although single study evaluation articles were identified from within their reference sections and were included if they met the search criteria. Also excluded were articles reporting findings for pre-service training activities.

Data collection and analysis continued in an iterative process, in which newly selected articles were read and coded, and re-read and re-coded as additional articles were retrieved. The retrieval and coding continued in an iterative process until the review process reached theoretical saturation (that is, no new categories or themes emerged) [21]. The final thematic analysis of training outcome reports was completed on a data set of 70 articles.

### Data analysis and framework development

#### Key informant interview data

Following completion of the interviews, transcripts created from the recordings and interviewer notes were systematically coded for thematic content independently by two reviewers. After an initial set of codes was completed, emerging themes were compared between the coders, and an iterative process of reading, coding and revising resulted in a final set of major themes identified from the transcripts. Representative quotations to illustrate each theme were excerpted and organized, and these final themes informed the development of the training evaluation framework.

#### Thematic analysis of published articles

Similar to the qualitative approach used to analyze the interview data, articles retrieved during the literature search were read by three analyst evaluators. Reported outcomes were systematically coded for themes, and those themes were then synthesized into a set of categories. The categories were compared among reviewers, re-reviewed and revised.

#### Development of the framework

The themes and categories that were identified in the analysis of data in the key informant interviews and the thematic analysis of training outcome reports were further integrated and synthesized. They were then compared to existing training evaluation models, to form an overall typology conceptualizing the relationships among all the prioritized elements. Finally, this information was used to form a new draft framework.

### Refinement and validation

The framework was then validated [25] through an iterative process using key informant and stakeholder feedback. Feedback was received from participants in the original key informant interviews, as well as from individuals new to the project. A total of 20 individuals, ranging from project managers and organizational administrators to professional evaluators, provided feedback on one or more draft versions of the model. After feedback was received, the model was revised. This cyclical process of revision, feedback and incorporation of revisions was repeated three times.

In addition, the framework was pilot-tested with two in-service training programs to verify its applicability in “real life.” For each pilot study, members of the training programs were coached on how to use the framework to describe anticipated outcomes and explore factors which may impact the evaluation. Within four weeks of their experiences with the draft framework materials, confidential feedback from pilot users was requested both in-person and by email. Users were asked to provide information on what worked well and what suggestions they might have to strengthen the framework and support its applicability in the field. This feedback was used to guide further improvement of the framework and accompanying materials.

## Results

Findings from the data analysis are first summarized below followed by a description of the Training Evaluation Framework, which was conceptualized and developed based on these findings.

### Data analysis: findings

#### Key informant interviews

The analysis of interview transcripts identified several major themes and sub-themes related to the development of a health care worker training outcome evaluation.

1) ***The lack of reporting on training outcomes is a gap in our current knowledge base.***

Interviewees acknowledged the existing gap related to reported training outcomes, and expressed a desire for it to be addressed. For example:

“We’re getting a lot of pressure from the PEPFAR side to link everything to health outcomes. . . . We need more monitoring and evaluation, but it may be that there’s not an easy way to get that. And it’s like this big thing that’s hard to address . . .”

“It would be nice to be able to prove that [in-service training] is worth the money.”

“Right now, clearly there’s not a lot of data to point to, to tell you we should put this amount of money into this versus that. How much should we put in pre-service? How much should we put in in-service?”

2) ***There are many challenges associated with successful evaluation of training outcomes.***

Interviewees discussed their perceptions regarding the many challenges associated with conducting evaluations of in-service training programs.

a. ***There is a lack of clear definition of what is meant by “training outcomes.”***

A lack of clarity on definitions, including what is meant by “outcome” and “impact,” was reported. Interviewees felt that addressing this challenge would help the overall move toward providing greater evidence to support training interventions. For example:

“It depends on what your end point is. If your end point is people trained, then you think it’s an outcome. If your end point is people treated, then that’s a different outcome. If your end point is people alive, then you’re going to have a different orientation.”

“I think we do have to broaden that definition [of training outcome]. If it really is incrementally first ‘service delivery improvement,’ and then, ‘health outcome improvement,’— to me [outcome evaluation requires] sort of a benchmarking approach.”

a. ***The contexts in which training evaluations occur are extremely complex.***

Frequently reported, for example, were the mobility of health care workers; lack of baseline data collected before interventions are begun; lack of infrastructure; and the fact that some communities or organizations may have multiple kinds of program interventions occurring simultaneously.

“Where there’s so much else going on, you can’t do an impact evaluation, for sure, on whether or not the training worked.”

“ . . . how are you going to attribute that it was the in-service training that actually increased or decreased performance? . . . How are we going to associate those two?—it’s hard to tease out.”

a. ***It is difficult to design an evaluation that demonstrates a link between a training intervention and its intended effects.***

Interviewees cited many potential methodological challenges and confounders to an evaluator’s ability to attribute an outcome, or lack of outcome, to a training intervention. These were discussed at the level of the individual trainee; in the context of the work sites to which trainees return and are expected to apply their new skills and knowledge; and at the larger population level, where health impacts might be seen. For example, at the individual level, interviewees indicated a number of issues that may impact training outcomes, including trainees’ background knowledge and experience, their life circumstances, and their motivation:

“ . . . who did you select [for the training]? What was their background? Were they the right person, did they have the right job, did they have the right education prior to walking in the door? Did they have an interest and a skill set to benefit from this training?”

At the facility or worksite level, trainees’ access to follow-up mentoring, management support, supplies, equipment and other infrastructure issues were noted by interviewees as impacting outcomes:

“People say, ‘I learned how to do this, but when I get back to my office they won’t let me do it. I’m not allowed to.’”

“Infrastructure - do they have facilities, do they have drugs, do they have transport? Are the policies in place for them to do the thing that they’re supposed to do?”

At a larger, health system and population level, a number of factors were suggested by interviewees as confounders to outcome evaluation, including supply chain issues, policies, pay scales and available community support resources.

“ . . . an individual gets trained and goes back to an office. . . . The policy that gives him licensure to do [what he was trained to do], that’s a system issue.”

a. ***Limited resources is an issue that impacts the ability of a program to conduct effective outcome evaluations****.*

For example, several interviewees cited limited funding and time for rigorous evaluation:

“I think it’s resources. To be able to do that kind of evaluation requires a lot of time and a lot of resources. And we just don’t have that.”

“I think the assumption for partners is, we can’t do that [training outcome evaluation]. We don’t have enough money. [But we should] identify ways to be able to do it most efficiently and to show that it is possible.”

3) ***A revised training evaluation framework would be useful if it included specific elements.***

a. ***A framework should show effects of interventions at several levels.***

In addition to suggestions related to evaluation levels, some participants described their wishes for what a training evaluation might include. Many expressed optimism that despite the complexities, evaluating outcomes is possible. Several interviewees spoke to the concept that there are different levels at which changes occur, and suggested that evaluation of outcomes should take these into consideration:

“Everything has your individual, organizational, and systemic level component to it, and it’s trying to link it - [not only] talking about capacity building itself, but its effect on health outcomes.”

a. ***A framework should support users to evaluate the complexity of the contexts in which training interventions occur.***

Incorporating a variety of methodologies into an evaluation, and exploring questions related to nuanced aspects of an intervention and its complex context were cited as important features of an improved evaluation approach:

“What we’re trying to understand is the things that go on inside. Between the point of the health training and the health outcome – what happens in the middle that makes it work or not work, or work better?”

“What different trainings are most effective, what is most cost-effective, these are the kinds of things, for this day and age, that we should be talking about.”

“It’s sort of you getting in there and doing more of the qualitative work… getting in there and understanding a little more richness about their environment.”

“I think evaluation will need to begin to look at how things become absorbed and become standardized in practice and in planning.”

In summary, analysis of interview transcripts revealed themes which included acknowledgement of the current gap in reporting training outcomes, and the perceived barriers to conducting training outcome evaluation, including limited resources available for evaluation, methodological challenges and the complex contexts in which these trainings occur. Interviewees also described hoped-for training evaluation elements, including integrating qualitative and quantitative methodologies and framing outcomes in a model that considers multiple levels, including individual, organizational and health systems.

#### Thematic analysis of published training outcome reports

The thematic analysis of 70 published articles identified several themes and categories for in-service training evaluation outcomes, and pointed to structured relationships among several levels and types of outcomes. The outcome categories included knowledge, attitude and skill changes; performance improvement; health impacts; and improvements made to organizational systems. The themes were then further sorted into three levels: individual, organizational and health systems/population. The final taxonomy, as well as the number of papers which reported outcomes in each category, is shown in Table 1.

**Table 1:** In-service training evaluation outcomes identified in thematic analysis of published articles reporting training outcomes

**Level**	**Category**	**Papers (#)**
Individual	Knowledge, attitude, skill	21
	Performance	30
	Patient outcomes	12
Organizational	Performance improvement	16
	Systems improvement	4
	Health outcomes	10
Health system/population	Performance improvement	9
	Systems improvement	3
	Health outcomes	11

At the individual level, outcomes were arranged by health care worker knowledge, attitude or skill; health care worker performance; and patient health outcomes. At the organizational level, papers were sorted into organizational performance improvements, system improvements and organizational-level health improvements; and at the health systems/population level, they were sorted by population-level performance improvements, system improvements and population-level health improvements.

These categories and levels were non-mutually exclusive; approximately half (34, 49%) of the papers reported outcomes in more than one outcome category. Citations, summaries of outcomes and outcome categories of these articles are included in Additional file 1. While not an exhaustive list of all published training evaluations, the findings from the thematic analysis of training outcome reports in the published literature provide ample evidence of the feasibility of implementing evaluations of training outcomes using a wide variety of research designs and methods.

### The training evaluation framework

#### Design and structure

The findings described above informed the development of a formalized Training Evaluation Framework, designed to serve as a practical tool for evaluation efforts that seek to link training interventions to their intended outcomes. The structure of the Framework includes major evaluation levels (individual, organizational and health systems/population), and is also designed to acknowledge the complexities involved in attributing observed outcomes and impacts to individual training interventions.

**Figure 1:**
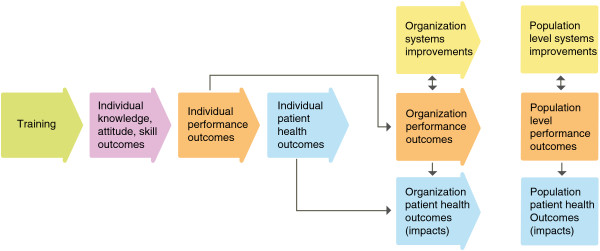
**Training evaluation framework skeleton.** Purple – Knowledge, attitude, skill outcomes. Orange – Performance outcomes. Yellow – Systems improvements. Blue – Patient health outcomes.

**Figure 2:**
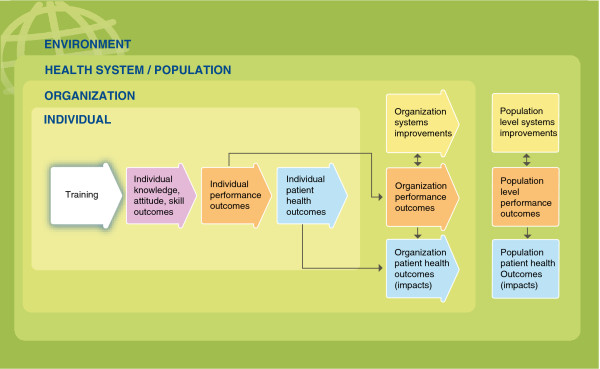
**Training evaluation framework with nested levels.** Purple – Knowledge, attitude, skill outcomes. Orange – Performance outcomes. Yellow – Systems improvements. Blue – Patient health outcomes. Three innermost green rectangles – nested levels of change. Darker, outermost green rectangle – environmental context.

**Figure 3:**
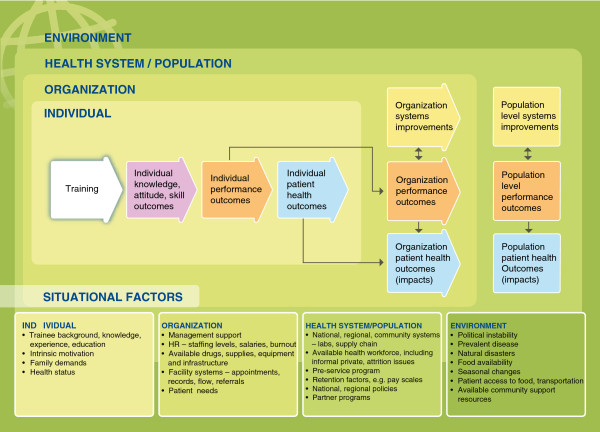
**Training evaluation framework with nested levels and situational factors.** Purple – Knowledge, attitude, skill outcomes. Orange – Performance outcomes. Yellow – Systems improvements. Blue – Patient health outcomes. Three innermost green rectangles – nested levels of change. Darker, outermost green rectangle – environmental context.

**Figure 4:**
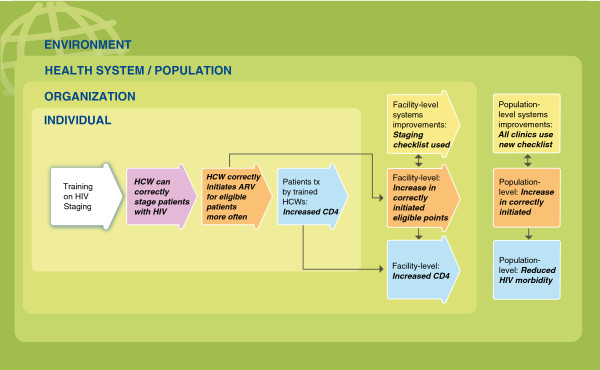
**Example of training evaluation framework for HIV clinical staging training.** Purple – Knowledge, attitude, skill outcomes. Orange – Performance outcomes. Yellow – Systems improvements. Blue – Patient health outcomes. Three innermost green rectangles – nested levels of change. Darker, outermost green rectangle – environmental context.

To help evaluators, implementers and other stakeholders internalize and use the Framework, the team created a series of graphics that visually demonstrate key concepts and relationships. In Figures 1, 2, 3 and 4, several of these graphics depict each type of training outcome and illuminate the relationships between outcomes seen at the individual, organizational and health systems/population levels. They also introduce other, “situational” factors that may influence a training outcome evaluation.

##### Training outcomes

The skeleton of the Framework, shown in Figure 1, includes the four types of training outcomes identified in the thematic analysis, coded by color. The purple box represents the most proximal outcome to health care worker training, in which improvements in participants’ content knowledge, attitude and skill are demonstrated. From these outcomes, and assuming necessary elements are in place, individual on-the-job performance improvements occur; these are shown in the orange boxes and can be measured at the individual, organization or population level. Systems improvements, shown in the yellow boxes, might also result from successful training interventions, and can be identified at the organizational or population level. Finally, the health improvements resulting from health care worker performance and systems improvements, which might be found at the individual, organizational or population level, are represented in the blue boxes.

##### Nested outcome levels

Figure 2 shows the logical flow of the Framework, which reflects the way outcome levels are, in practice, “nested” within one another. Individual level outcomes, set to the middle left and shaded in the lightest green, are nested within the organizational level (darker green), which sits within the larger health system and population level. In addition, the Framework recognizes that these levels exist within a larger, environmental context, which might include, for example, seasonal climate conditions, food security issues and political instability. These nested levels are an element of many capacity building models [23], and several interviewees suggested that they should be integrated into the framework. The structure also reflects findings from the thematic analysis of published articles reporting training outcomes, which suggest that outcome evaluations tend to focus on the individual, organizational/system or population levels, respectively.

##### Situational factors

The levels are important when considering the logical progression of outcomes resulting from training. They also frame another important issue included in the Framework: “situational factors,” or confounders, which are exogenous to the training intervention itself but could strongly influence whether it achieves its desired outcome. Figure 3 shows examples of situational factors, placed in bulleted lists. These factors are not an exhaustive list of the possible mitigating factors, but they provide examples and general types that should be considered. The factors are presented at the levels where they would most likely influence the desired training outcomes.

### Applying the framework

During development, feedback was obtained from key informants regarding how the Framework might be used to evaluate the outcomes of specific trainings. One example provided was that of training on HIV clinical staging for health care workers in a district-level facility. The potential application of the Framework to this training is used as an example below, and illustrated in Figure 4.

#### Individual level

In Figure 4, the training intervention on HIV staging is shown in the white arrow on the left side of the graphic. Moving from left to right, an evaluator would consider the first outcome arrow, shown in purple. This arrow reflects individual changes in health care workers’ knowledge, attitude and skills resulting from the training. In this example, a skills assessment given after the training shows that the trainees can now correctly stage patients living with HIV; their skills have improved.

The second arrow, in orange, indicates that when the trainees are observed in their workplaces by an expert clinician, their staging matches the staging performed by the expert clinician, meeting an acceptable standard of competency. The trainees also initiate antiretroviral treatment for eligible patients more often than they did before the training. This reflects improvement in the workers’ on-the-job performance.

The third outcome arrow, in blue, illustrates patient health outcomes. In this example, patient medical records show that patients who are treated by the trained health care workers have higher CD4 counts than the patients of health care workers who have not attended the training. Thus, the health of the patients treated by the trained health care workers has improved.

#### Facility or organizational level

The first yellow arrow shows a facility-level system improvement: Following the training on staging, the facility initiates a new system in which a checklist is used for staging patients. In addition, facility records show an increase in patients correctly initiated on antiretroviral therapy. This is an organization-level performance outcome, shown in orange. As a result of this performance outcome there is also a facility-level patient health outcome: an increase in patient CD4 cell counts. This is shown in the blue box. Finally, an evaluator might also see similar improvements in systems, performance and overall health outcomes at the health system/population level. These are indicated in the yellow, orange and blue boxes to the far right.

Situational factors of particular relevance for this example (not pictured) might include the adequacy of antiretroviral treatment medication supplies and the functionality of laboratory equipment.

### Validation

In keeping with an emphasis on making the Framework practical and usable for experienced program evaluators, training program implementers and funders, additional evaluation planning tools were developed to accompany the Framework. These materials are collectively called the Training Evaluation Framework and Tools (TEFT). A website, http://www.go2itech.org/resources/TEFT, was developed to introduce the Framework and tools and guide evaluators to use them effectively. It includes a link for users to provide feedback and suggestions for improvement.

Validation of the Framework included a cyclical process of development, feedback and revision. Pilot users suggested that the model responded well to their needs. Individual team members from two training programs that have accessed and used the TEFT for evaluation planning provided positive feedback regarding the model’s usability and value:

“The process was helpful in thinking through the whole project - not just thinking about the one thing we needed for our next meeting. Slowing down to use the tools really helped us think, and then, when the plan for the training changed, we had put so much time into thinking that we could really handle the changes well.”

“Going through the Training Evaluation Framework helped us to think through all of the different outcomes of our training program, how they are related to each other, and how that can contribute to our ultimate impact. It really helped me to see everything together and think about the different factors that could influence the success of our training program.”

## Discussion

In limited-resource settings, especially those that have been heavily impacted by the HIV epidemic, there is likely to be a continued reliance on in-service training as a strategy to update the skills of health care workers and address the changing needs of health systems. Strengthening in-service training and pre-service training programs will be particularly important as the HIV/AIDS epidemic changes; as improved prevention, care and treatment strategies are discovered; and as national and global funding priorities evolve.

Stakeholders involved in these training efforts increasingly want to know what public health impact can be shown in relation to the millions of training and retraining encounters supported globally. However, demonstrating the linkages between in-service training and patient- and population-level outcomes can be a daunting challenge. An important first step in evaluating any intervention, including training, is to describe exactly what is being evaluated and what its intended result is, and to accurately identify potential confounders to assessing training effectiveness. The Training Evaluation Framework described in this article is intended to serve as a useful and practical addition to existing tools and resources for health and training program managers, funders and evaluators.

This framework shares a number of elements with other training evaluation models, including its basic “if-then” structure, and a series of hierarchical categories that build upon one another. The Training Evaluation Framework described here expands upon three of Kirkpatrick’s four levels of training evaluation (Learning, Behavior and Results) [16,17], as well as the modifications proposed by others [18-21], taking into account the context in which outcomes can be seen and guiding users to identify potential confounders to the training evaluation. The Framework adds value to existing frameworks by focusing specifically on health care worker training and explicitly addressing the multiple levels at which health training outcomes may occur.

Although the Framework articulates a theoretical causal linkage between the individual, organizational and population level, this does not presuppose that evaluation must occur at each point along the evaluation continuum. In fact, it is extremely rare for evaluations to have the resources for such exhaustive documentation. Rather, the Framework should guide training program implementers and others in thinking about available resources, existing data and the rationale for evaluating outcomes at particular points along the continuum. Once this has been determined, a variety of evaluation research designs (including but not limited to randomized controlled trials) and methods can be developed and implemented to answer specific evaluation questions [7,26].

The Training Evaluation Framework has commonalities with methodological approaches that have been proposed to address the complexities inherent in evaluating program interventions implemented in non-research settings. For example, both Realist Evaluation [27] and Contribution Analysis [28,29] incorporate contextual factors into an evaluation framework. Both of these evaluation approaches acknowledge that in many instances, an intervention’s contribution to a particular outcome can be estimated but may not be proven. In Contribution Analysis, contextual factors must be considered in analyzing the contribution of the intervention to the outcome observed. Context analysis is also an essential component of Realist Evaluation, in which the underlying key questions are: What works, for whom, in what circumstances, in what respects, and why? Realist Evaluation suggests that controlled experimental design and methods may increase the degree of confidence one can reasonably have in concluding an association between the intervention and the outcome, but suggests that when contextual factors are controlled for, this may “limit our ability to understand how, when, and for whom the intervention will be effective” [27]. Similarly, Contribution Analysis speaks to increasing “our understanding about a program and its impacts, even if we cannot ‘prove’ things in an absolute sense.” This approach suggests that “we need to talk of reducing our uncertainty about the contribution of the program. From a state of not really knowing anything about how a program is influencing a desired outcome, we might conclude with reasonable confidence that the program is indeed . . . making a difference” [28]. Both Realist Evaluation and Contribution Analysis emphasize the importance of qualitative methods in order to identify and better understand contextual factors which may influence evaluation results.

### Limitations

As is common to all qualitative inquiry, the results of this inductive approach are influenced by the experiences and perspectives of the researchers [30]. To ensure broad applicability of the framework, validation measures were undertaken, including triangulation of the data sources, obtaining feedback from stakeholders, and “real-world” testing of the framework. Finally, as with any framework, the usefulness of the Training Evaluation Framework will depend on its implementation; it can help to point users towards indicators and methods which may be most effective, but ultimately the framework’s utility depends upon quality implementation of the evaluation activities themselves.

## Conclusion

The Training Evaluation Framework provides conceptual and practical guidance to aid the evaluation of in-service training outcomes in the health care setting. It was developed based on an inductive process involving key informant interviews, thematic analysis of training outcome reports in the published literature, and feedback from stakeholders, and expands upon previously described training evaluation models. The framework guides users to consider and incorporate the influence of situational and contextual factors in determining training outcomes. It is designed to help programs target their outcome evaluation activities at a level that best meets their information needs, while recognizing the practical limitations of resources, time frames and the complexities of the systems in which international health care worker training programs are implemented.

Validation of the framework using stakeholder feedback and pilot testing suggests that the model and accompanying tools may be useful in supporting outcome evaluation planning. The Framework may help evaluators, program implementers and policy makers answer questions such as, what kinds of results are reasonable to expect from a training program? How should we prioritize evaluation funds across a wide portfolio of training projects? What is reasonable to expect from an evaluator in terms of given time and resource constraints? And, how does the evidence available for my training program compare to what has been published elsewhere? Further assessment will assist in strengthening guidelines and tools for operationalization within the health care worker training and evaluation setting.

## Abbreviations

PEPFAR: The United States president’s emergency plan for AIDS relief; TEFT: Training evaluation framework and tools.

## Competing interests

The authors declare they have no competing interests.

## Authors’ contributions

GO developed the initial concept and participated in key informant interviews, data analysis, framework development and the writing of the manuscript. TP participated in development of the initial concept, key informant interviews and the writing of the manuscript. FP participated in key informant interviews, data analysis, framework development, literature review and writing of the manuscript. All authors read and approved the final manuscript.

## Supplementary Material

Additional file 1 **TEFT literature summaries.** Citations, summaries of outcomes and outcome categories of 70 articles reviewed during development of the Training Evaluation Framework. Click here for file
